# Terminal deletion of chromosome 13 in a fetus with normal NIPT: The added value of invasive prenatal diagnosis in the NIPT era

**DOI:** 10.1002/ccr3.2889

**Published:** 2020-05-13

**Authors:** Evy Vervecken, Bettina Blaumeiser, Tina Vanderheyden, Jan Hauspy, Katrien Janssens

**Affiliations:** ^1^ Department of Obstetrics and Gynaecology GZA Hospitals St. Augustinus Wilrijk Belgium; ^2^ Center of Medical Genetics University Hospital and University of Antwerp Antwerp Belgium

**Keywords:** amniocentesis, deletion chromosome 13, holoprosencephaly, invasive prenatal diagnosis, NIPT, noninvasive prenatal test

## Abstract

In the age of noninvasive prenatal testing, there is still an important role for invasive prenatal diagnosis, even for chromosomes 13, 18, and 21.

## Graphical abstract

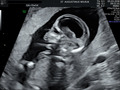

## 1 INTRODUCTION

Noninvasive prenatal testing is used for the screening of trisomy 13, 18, and 21. Nevertheless, when ultrasound abnormalities are present, invasive prenatal testing is still the preferred test. We report a case of fetal holoprosencephaly in which a pathogenic deletion on chromosome 13 was missed with NIPT.

Terminal deletions of chromosome 13 represent a rare genetic disorder causing a variety of congenital defects. The phenotype depends on the size of the deleted region and the grade of mosaicism (if present). Common clinical symptoms comprise mental retardation, growth restriction, facial dysmorphisms, and malformations of brain, eyes, kidneys, and heart.^1^, ^2^, ^3^ This syndrome was first described in the 60s by Allderdice et al among others.^1^ The phenotype may vary depending on the deleted region.^3^

Routine obstetric care in Belgium includes offering a noninvasive prenatal test (NIPT) as a first‐tier screening for common aneuploidies. Ultrasound screening should be performed before. If abnormalities are seen, it is recommended to proceed with invasive diagnostics immediately instead of the NIPT.^4^, ^5^, ^6^

In this paper, we present a case of fetal holoprosencephaly in which an aneuploidy of chromosome 13 was suspected, and a NIPT was performed because of the technical impossibility to carry out chorionic villi sampling (CVS) at an early stage of the gestation.

## 2 CASE REPORT

**Figure 1:**
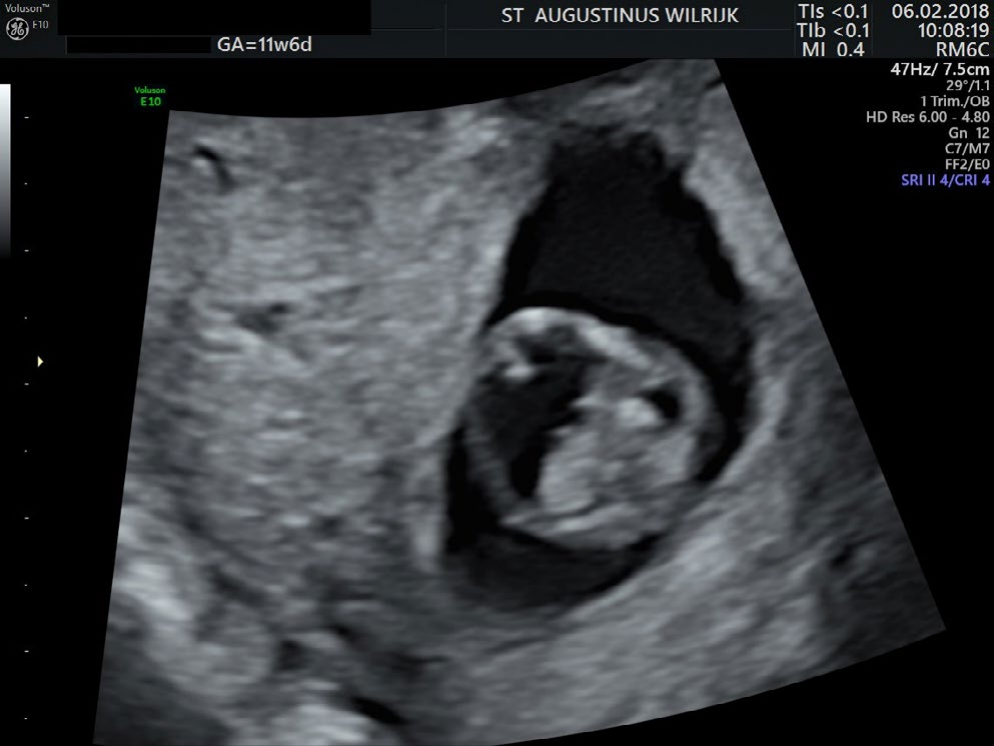
Ultrasound at 11 wks and 6 d shows an abnormal cerebral anatomy, with preferential diagnosis of holoprosencephaly

A 32‐year‐old woman presented for the first time at 11 weeks of gestation. Ultrasound showed an abnormal cerebral anatomy, with preferential diagnosis of holoprosencephaly (Figure 1). Crown‐rump length (CRL) was under the 5th percentile.

The woman was a primigravida in good general health. The father was a 36‐year‐old man without previous health problems. The couple was of North African origin, not consanguineous, and conception was spontaneous.

Because of the fetal cerebral anomaly, the couple was counseled for a chorion villus sampling (CVS). Inaccessibility of the placenta precluded the CVS and the obstetrician together with a clinical geneticist decided to proceed with a NIPT. The NIPT applies shallow whole‐genome sequencing to detect not only trisomy of the most common chromosomes (13/18/21), but also aneuploidies of the other autosomes and the sex chromosomes, using a modified version of the pipeline described by Bayindir et al^7^ Depending on the size, chromosomal location, and fetal fraction, the NIPT can also detect fetal subchromosomal aberrations. The NIPT did not show any aneuploidy or other large anomalies in this female fetus; the fetal fraction, measured using SeqFF,^8^ was 5.1%.

At 12 weeks, before receiving the result of the NIPT, the couple was seen by the pediatric neurologist and was counseled about the poor prognosis of the child, especially regarding psychomotor development. The parents were offered a termination of the pregnancy but they decided to continue the gravidity citing religious reasons.

**Figure 2:**
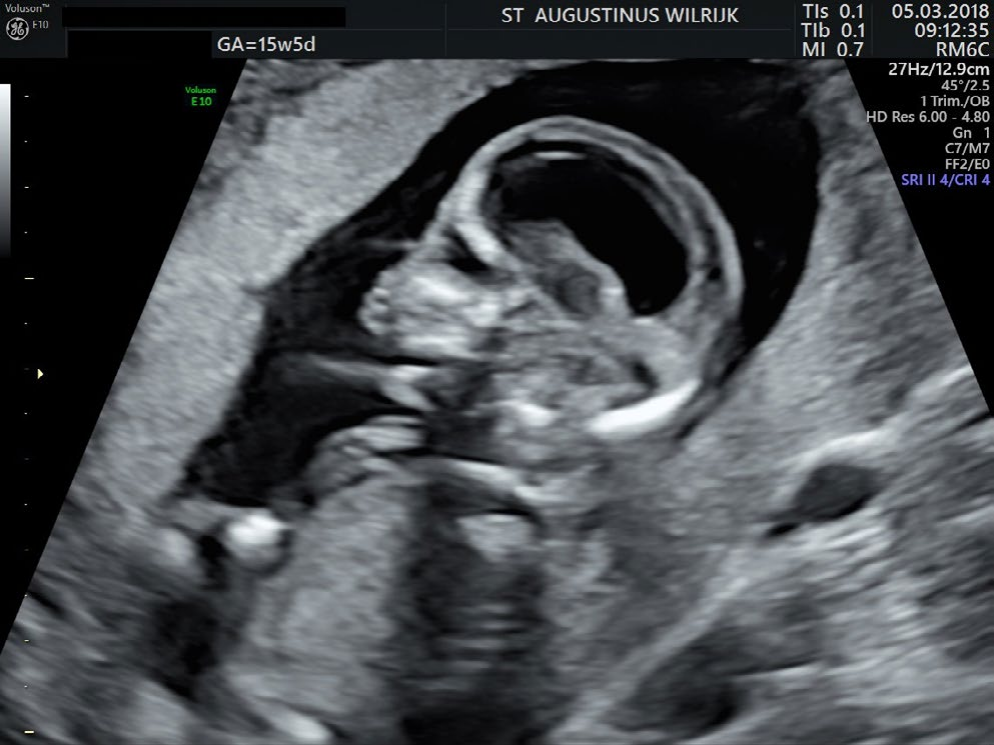
Ultrasound at 15 wks and 5 d shows a more pronounced holoprosencephaly

**Figure 3:**
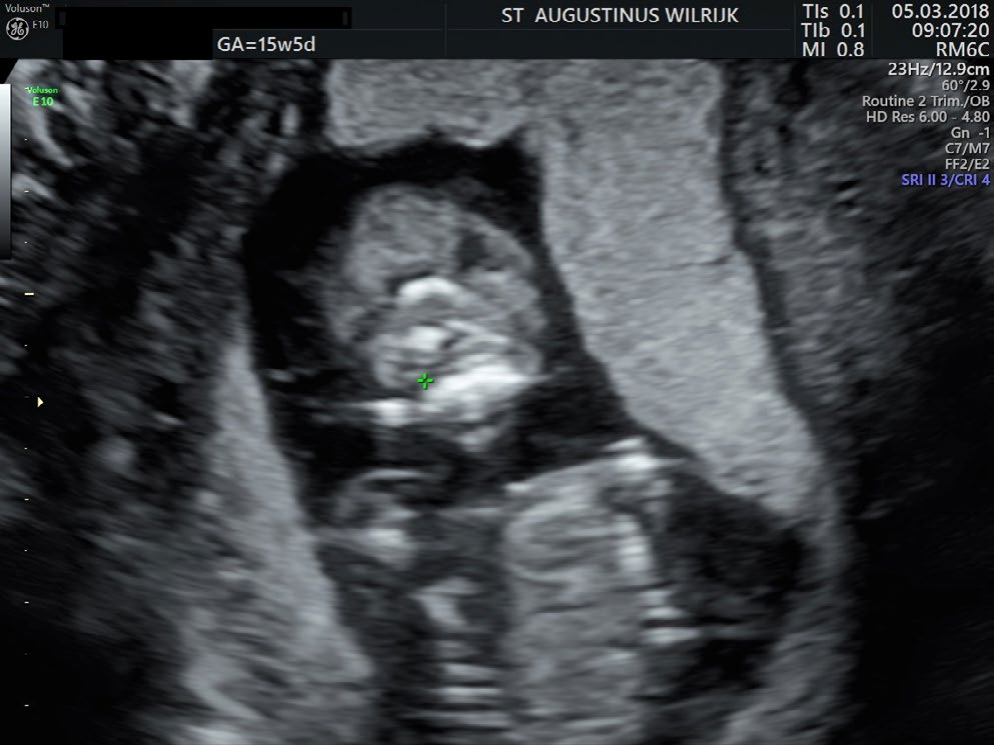
Ultrasound at 15 wks and 5 d shows the impression of a unilateral cleft lip

Despite the normal NIPT result, an amniocentesis was performed at 15‐week pregnancy. During the associated expert ultrasound several abnormalities were seen: a more pronounced holoprosencephaly (Figure 2), an abnormal posterior fossa with the suspicion of a Dandy‐Walker malformation, and the impression of an unilateral cleft lip (Figure 3) and overriding fingers.

The amniotic fluid was investigated using SNP array, a genome‐wide technology detecting genomic aberrations down to 400 kb using the CNV‐Webstore software for CNV analysis.^9^ It showed a nonmosaic 32 Mb deletion on the long arm of chromosome 13: arr 13q31.1q34(82446327_115106996)x1. Deletions of this region have been described in patients with mental retardation, facial dysmorphy, and brain, eye, renal, and cardiac malformations.^1^, ^2^, ^3^ The deletion encompasses the *ZIC2* gene; haploinsufficiency of this gene is known to result in holoprosencephaly.^10^ In conclusion, the fetal phenotype was in concordance with the genetic findings. On reinspection of the NIPT profile, a deletion of the long arm of chromosome 13 was indeed suspected. Bin‐based reanalysis of this region showed an aberrant pattern for 63 consecutive 50 kb bins, corresponding to a region of 32.5 Mb (83500000‐115050000), which is in almost perfect concordance with the array findings. The regional *Z*‐score reached −5.97, which is comparable to *Z*‐scores for other proven fetal copy number variations (CNVs). The read ratio, representing the normalized number of reads in a particular region, was 0.98, well within the normal range for a diploid region; this can, however, be explained by the low fetal fraction (5.1%).

A follow‐up ultrasound at 17 weeks of pregnancy clearly showed a unilateral facial cleft as well as a more pronounced holoprosencephaly with a Dandy‐Walker malformation and abnormal positioning of feet and knees. Based on the bad prognosis and the proven genetic aberration, the couple now chose to terminate the pregnancy. The termination was performed at 18 weeks of pregnancy after the approval of the local ethical committee of the hospital.

**Figure 4:**
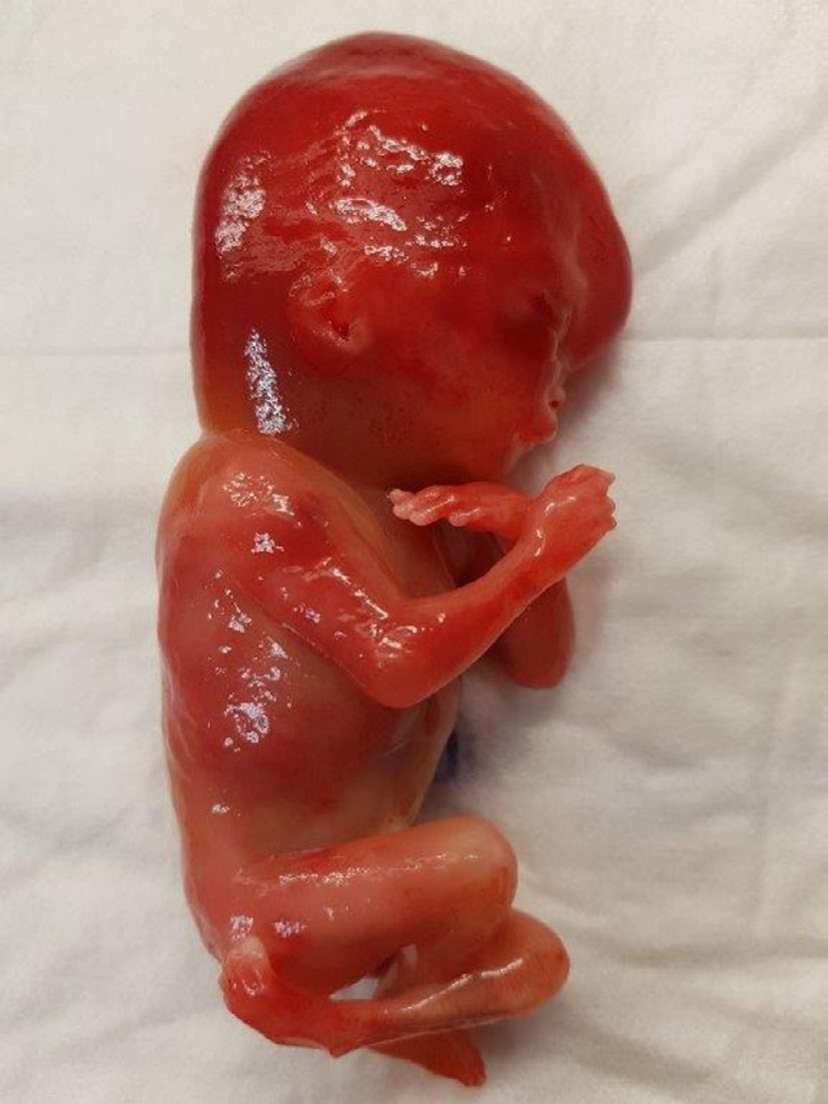
Fetus of 18 wks with a low‐set implantation of the ears, fixed adduction of the ankles, and an increased flexion of the forefeet

**Figure 5:**
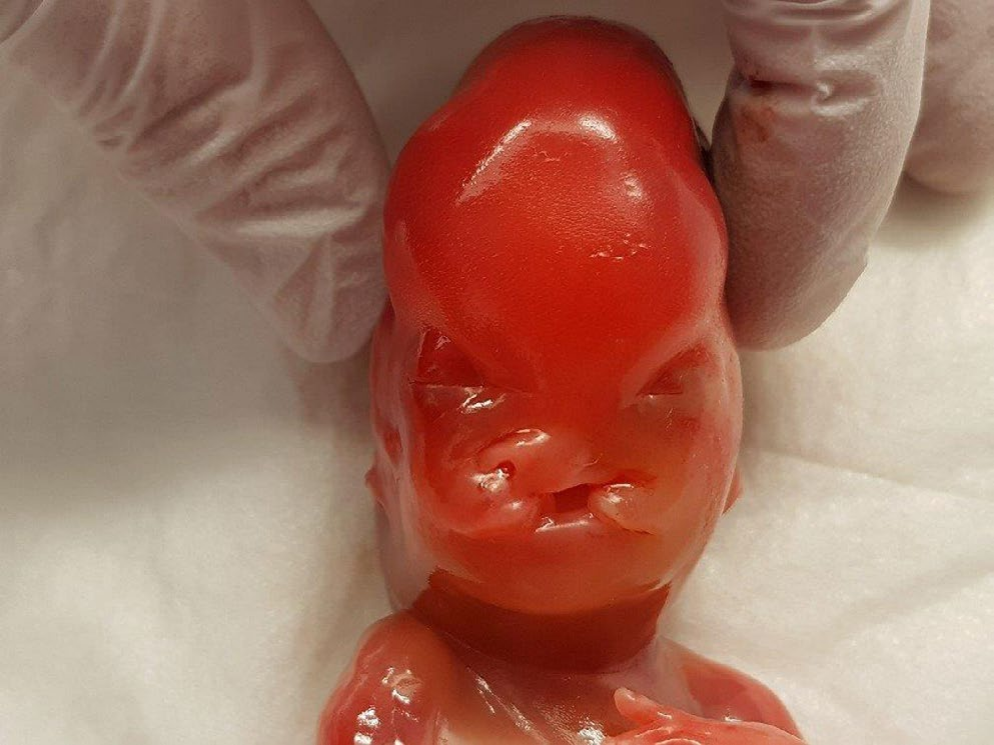
Fetus of 18 wks with a cleft lip and hypertelorism

**Figure 6:**
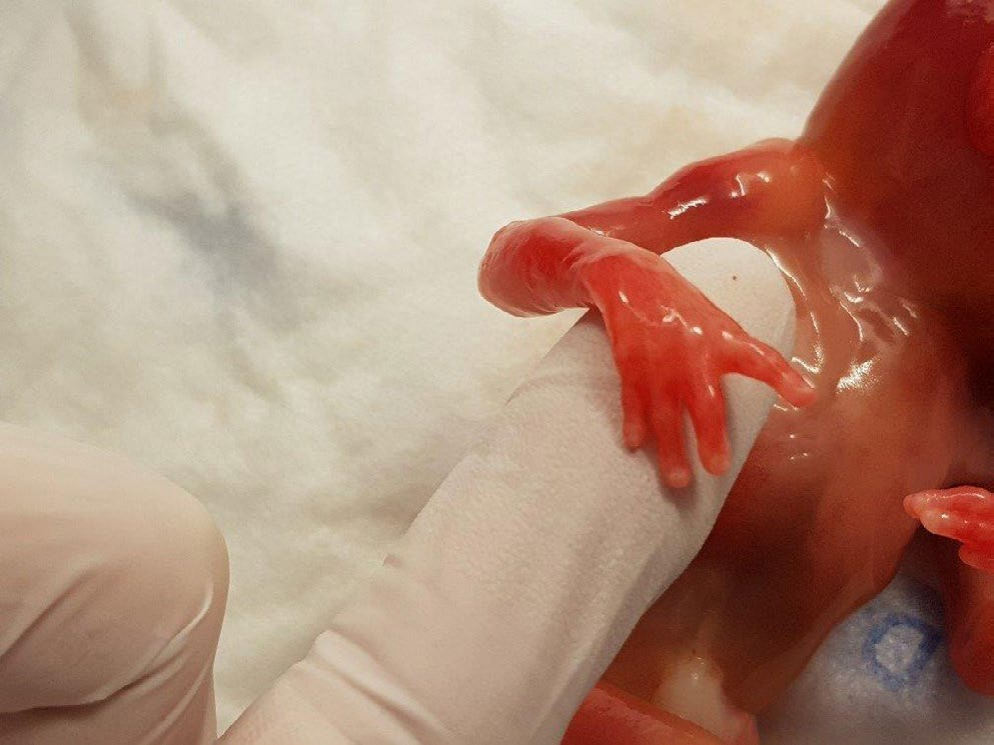
Fetus of 18 wks with absent right thumb

On autopsy, the female fetus presented with a below‐average birthweight of 115 grams (average at 18 weeks is 194 ± 65 g). The head circumference (12.5 cm) was high in comparison with the other measurements of the body. Dysmorphic facial features included unilateral cleft lip and palate, low‐set implantation of the ears (Figure 4), hypertelorism, and neck edema (Figure 5). There were both upper and lower limb abnormalities: The right thumb was absent (Figure 6), the left was only rudimentary, the ankles showed a fixed adduction, and the forefeet demonstrated an increased flexion (Figure 4).

X‐ray of the fetal skeleton showed only four metacarpals on both hands and missing phalanges of the thumbs. On the left hand, a soft tissue component, compatible with a rudimentary thumb, was visible.

Macro‐ and microscopic evaluation of the placenta were normal.

Karyotyping of both parents, performed to exclude translocations as the basis of the fetal genetic aberration, was normal.

## 3 DISCUSSION

Since July 2017, the NIPT is reimbursed in Belgium for all pregnant women, regardless of their a priori risk. Recommendations are to perform the NIPT only after normal findings on ultrasound at 12 weeks. In case of abnormal ultrasound findings, it is recommended to proceed immediately to invasive diagnostics.^4^, ^5^, ^6^

NIPT investigates cell‐free DNA (cfDNA) from apoptotic cells of both maternal and fetal origin that shed their DNA in the maternal bloodstream. The fetal component, the cell‐free fetal DNA (cffDNA), is derived from the placenta (the trophoblast cell layer)^11^; the fetal fraction (ff), the percentage of cfDNA that is fetal in origin, shows a median of 10% at 10‐week gestational age.^12^

The sensitivity of NIPT to detect the common aneuploidies has been illustrated in numerous papers, both in high‐risk and low‐risk populations (see Gil et al^13^ for a meta‐analysis). However, despite being a screening test with a highly superior sensitivity and specificity over biochemical prenatal screening tests like the triple test, NIPT for aneuploidies should not be considered a diagnostic test. Several factors, mostly biological in origin, can lead to false‐negative results. The most important of them is fetal fraction: In general, 4% ff is considered to be the cut‐off for a reliable NIPT result. Consequently, using a reliable method to measure ff is of high importance. In our laboratory, the SeqFF method has been adopted; it shows a Pearson correlation of 0.921 when compared to an SNP‐based approach, which is considered to be the most accurate method for ff determination. In our laboratory, correlation with the ff based on Y‐reads in boys was also high (0.907). Second, true fetal mosaicism (type 5) can lead to false‐negative results as well as the placenta, and consequently, the NIPT result is euploid, while the fetus is not.

The sensitivity of NIPT to microdeletions on the other hand remains poorly investigated. Some commercial laboratories routinely offer an extended NIPT including some microdeletions, but two main problems remain.

1. Validation studies have mostly been performed on artificial samples; the low prevalence of the individual microdeletion syndromes hampers the inclusion of large numbers of “real” samples. For the 22q11.2 deletion, the most common microdeletion, a recent review,^14^ shows a detection rate of 66.7%‐90% on a total of 20 samples with a fetal 22q11.2 deletion, far too little to draw any conclusions on the sensitivity in the general population. Moreover, none of the studies are prospective. Numbers for other, rarer microdeletion syndromes are even smaller. Because of their rarity, positive predictive values are unacceptably low.
2. Companies try to convince patients and prescribers by stating the high combined incidence of microdeletions/duplications in all‐risk women (1/1000), but in reality, they offer testing for just a fraction of those and not necessarily those with the highest incidence. For example, one provider offers testing of the 16p12 syndrome, with an incidence of <1/1 000 000. Most professional organizations like the American College of Medical Genetics (ACMG), the International Society for Prenatal Diagnosis (ISPD), the American College of Obstetricians and Gynecologists (ACOG), and the American and European Society of Human Genetics (ASHG/ESHG) are at present not in favor of testing for microdeletions or at least want testing to be limited to clinically significant disorders with a well‐defined severe phenotype.

In Belgium, all eight genetic centers perform a NIPT based on shallow whole‐genome sequencing, allowing not only for the detection of the 3 common aneuploidies, but also of rare autosomal trisomies (RATs), maternal subchromosomal aberrations, and, in some cases, large, noncryptic fetal aberrations throughout the genome. Although the cutoff for the detection of fetal aberrations is considerably larger in size than most common microdeletions, our own experience and published reports have clearly shown that the sensitivity for the detection of these microscopic fetal aberrations is still much lower than for aneuploidies. Several biological factors contribute to this lower sensitivity:

1. The size of the aberration: The larger the aberration, the more clearly it will affect the overall *Z*‐score of the chromosome, triggering further investigations into a specific chromosome/region;
2. The fetal fraction: For trisomy 21, a very high correlation has been shown between fetal fraction and *Z*‐score (^15^ and own observations); it is plausible to assume that this is also the case for subchromosomal aberrations;
3. The chromosomal location: Some chromosomes are “noisier” than others, especially those with a high GC content, making it more difficult to pick up small variations.

Furthermore, technical issues are of importance as well:

1. The sequencing depth: The more reads are generated within a given fragment, the higher the chance that a deviation from a reference set of euploid samples will be picked up.
2. The overall quality of the sample will determine the certainty with which CNVs can be called; samples of lower quality will be “fragmented” and might contain dozens of apparent fetal aberrations.

In conclusion, different factors can contribute to a false‐negative result for fetal subchromosomal aberrations. Most of these could be circumvented by much deeper sequencing than is common in genome‐wide NIPT, but this would increase the cost substantially, making it a test that cannot be offered to all pregnant women. Since all women, regardless of their age, have the same a priori risk of carrying a fetus with a subchromosomal aberration (with the exception of women who are themselves (or their partner) carrier of a structural variant, eg, a balanced translocation), it is not opportune to offer an “extended” NIPT to a subset of pregnant women. In Belgium, where the reimbursement of NIPT is restricted to €260, it is financially not feasible to offer an extended NIPT detecting noncryptic (let alone cryptic) fetal aberrations in all pregnancies.

In a recent paper, NIPT could detect three out of three CNVs larger than 20 Mb, but differences in size between noninvasive and invasive testing were as high as 17 Mb.^16^ In a paper on approximately 10 000 cases undergoing genome‐wide cfDNA screening with a resolution of 7 Mb, 84 cases (0.82%) showed a subchromosomal CNV, but no follow‐up was performed, making it impossible to determine specificity and sensitivity for these aberrations.^17^ An earlier study from the same company^18^ showed one false‐negative and one false‐positive results in 43 cases with either a RAT, a microdeletion, or a microscopic aberration. However, a minimum of 32 mol/L reads was generated, and this was increased to 226 mol/L reads in case of discordancy—this is not realistic if NIPT is to be offered as a population screening. A study by Benn and Grati,^19^ looking at a large dataset of analyses on chorion villi biopsies under the assumption that NIPT would identify a similar spectrum of abnormalities with the same sensitivity as karyotyping, showed that in an all‐risk group of 100 000 pregnant women, 82 (0.082%) would show a nonmosaic clinically relevant subchromosomal aberration, of which 50% would occur in fetuses with an abnormal ultrasound. The Dutch TRIDENT study shows subchromosomal aberrations in 6/2527 cases (0.24%). All aberrations were confirmed, either in the amniotic fluid or in the cord blood. Two had multiple congenital anomalies, and one died in utero.^20^ A paper describing the percentage of confirmed fetal aberrations found by Belgian genetic centers in the first 2 years of NIPT reimbursement is under preparation.

If the case described here had followed our standard procedure, an invasive diagnostic procedure would have been the preferred choice. However, because of the inability to perform a CVS, a NIPT was done instead notwithstanding the presence of ultrasound anomalies. In spite of the normal NIPT result, an amniocentesis was performed because of the presence of major structural anomalies and the lack of an etiological diagnosis. This revealed a 32 Mb deletion which, based on the literature and concordance with the fetal phenotype, was classified as pathogenic. In retrospect, the presence of the deletion could have been suspected based on the plot of chromosome 13. However, our pipeline (which is based on the pipeline published by Bayindir et al^7^ with some modifications, eg, the calling of maternal CNVs and sex chromosomal aneuploidies) does not automatically call fetal aberrations. For every “aberrant” region of at least 400 kb (850 kb bins), a *Z*‐score and read ratio are calculated, but unless the CNV is clearly maternal in origin (read ratio close to 0.5 for a deletion or 1.5 for a duplication), these regions are only investigated if the chromosome plots are aberrant on visual inspection.

Determining the correct *Z*‐score and read ratio thresholds for accurate identification of fetal aberrations remains very difficult because of the lack of a substantial validation set. The read ratio correlates with the fetal fraction: A fetal fraction of 4% would cause the read ratio to change to 0.98 (deletion) or 1.02 (duplication), a variation that is seen numerous times throughout the genome of every sample. A higher fetal fraction (>10%) would change the read ratio to <0.95 or >1.05 for deletions and duplications, respectively, which would be easier to pick up. In this particular case, the read ratio was 0.98, well in concordance with the fetal fraction of 5.1%. Although suggestive of a deletion, the regional *Z*‐score of −5.97 is not convincing, given the size of the CNV; similar scores have been seen with false‐positive (unconfirmed) fetal CNVs, especially in cases of lower quality. An invasive follow‐up of all these NIPT cases is not acceptable, as it would reverse the trend of the decrease in prenatal invasive testing that was the result of the implementation of NIPT.

This case report illustrates that NIPT remains a screening test and is not suited to replace invasive prenatal diagnostics. NIPT results always have to be interpreted in the context of ultrasound findings, and a structural first‐trimester ultrasound should always proceed the NIPT sampling.

## CONFLICT OF INTEREST

None declared.

## AUTHOR CONTRIBUTIONS

BB, EV and TVH were involved in patient management. KS provided molecular analysis of samples. EV, KJ, TVH, and BB wrote the manuscript. JH was the local supervisor of the residents.
